# Statistical techniques used in analysing simultaneous continuous glucose monitoring and ambulatory electrocardiography in patients with diabetes: A systematic review

**DOI:** 10.1371/journal.pone.0269968

**Published:** 2023-02-24

**Authors:** Beatrice Charamba, Aaron Liew, Asma Nadeem, John Newell, Derek T. O’Keeffe, Timothy O’Brien, William Wijns, Atif Shahzad, Andrew J. Simpkin

**Affiliations:** 1 School of Mathematical and Statistical Sciences, National University of Ireland, Galway, Ireland; 2 Insight Centre for Data Analytics, National University of Ireland, Galway, Ireland; 3 The Lambe Institute for Translational Medicine, Curam and the Smart Sensors Lab, National University of Ireland, Galway, Ireland; 4 Department of Medicine, Portiuncula University Hospital, Saolta University Healthcare Group, Galway, Ireland; 5 Centre for Diabetes, Endocrinology and Metabolism, University Hospital Galway, Galway, Ireland; 6 Regenerative Medicine Institute, National University of Ireland, Galway, Ireland; Instituto Politécnico de Santarém: Instituto Politecnico de Santarem, PORTUGAL

## Abstract

**Objectives:**

There has been a steady increase in the number of studies of the complex relationship between glucose and electrical cardiac activity which use simultaneous continuous glucose monitors (CGM) and continuous electrocardiogram (ECG). However, data collected on the same individual tend to be similar (yielding correlated or dependent data) and require analyses that take into account that correlation. Many opt for simplified techniques such as calculating one measure from the data collected and analyse one observation per subject. These simplified methods may yield inconsistent and biased results in some instances. In this systematic review, we aim to examine the adequacy of the statistical analyses performed in such studies and make recommendations for future studies.

**Research questions:**

What are the objectives of studies collecting simultaneous CGM and ECG data? Do methods used in analysing CGM and continuous ECG data fully optimise the data collected?

**Design:**

Systematic review.

**Data sources:**

PubMed and Web of Science.

**Methods:**

A comprehensive search of the PubMed and Web of Science databases to June 2022 was performed. Studies utilising CGM and continuous ECG simultaneously in people with diabetes were included. We extracted information about study objectives, technologies used to collect data and statistical analysis methods used for analysis. Reporting was done following PRISMA guidelines.

**Results:**

Out of 118 publications screened, a total of 31 studies met the inclusion criteria. There was a diverse array of study objectives, with only two studies exploring the same exposure-outcome relationship, allowing only qualitative analysis. Only seven studies (23%) incorporated methods which fully utilised the study data using methods that yield the correct power and minimize type I error rate. The rest (77%) used analyses that summarise the data first before analysis and/or totally ignored data dependency. Of those who applied more advanced methods, one study performed both simple and correct analyses and found that ignoring data structure resulted in no association whilst controlling for repeated measures yielded a significant relationship.

**Conclusion:**

Most studies underutilised statistical methods suitable for analysis of dynamic continuous data, potentially attenuating their statistical power and overall conclusions. We recommend that aggregated data be used only as exploratory analysis, while primary analysis should use methods applied to the raw data such as mixed models or functional data analyses. These methods are widely available in many free, open source software applications.

## Introduction

Technological developments in recent years have allowed researchers and clinicians active in the field of endocrinology to use monitors to collect glucose measurements regularly over time. Continuous glucose monitors (CGM) measure interstitial glucose (IG) at regular intervals (e.g. every 5 minutes) giving superior granularity of data to traditional finger stick blood glucose measurement. With the introduction of economically friendly and accurate continuous electrocardiogram (ECG) monitors, data can now be collected on CGM and ECG simultaneously to study the relationship between glucose and the electrical activity of the heart. Studies utilising concomitant CGM and ECG data are becoming more prevalent, parallel to the increasing uptake of these technologies among people with diabetes, in particular, the CGM, which has been shown to be beneficial compared to self-monitoring of blood glucose [1–6].

**Fig 1:**
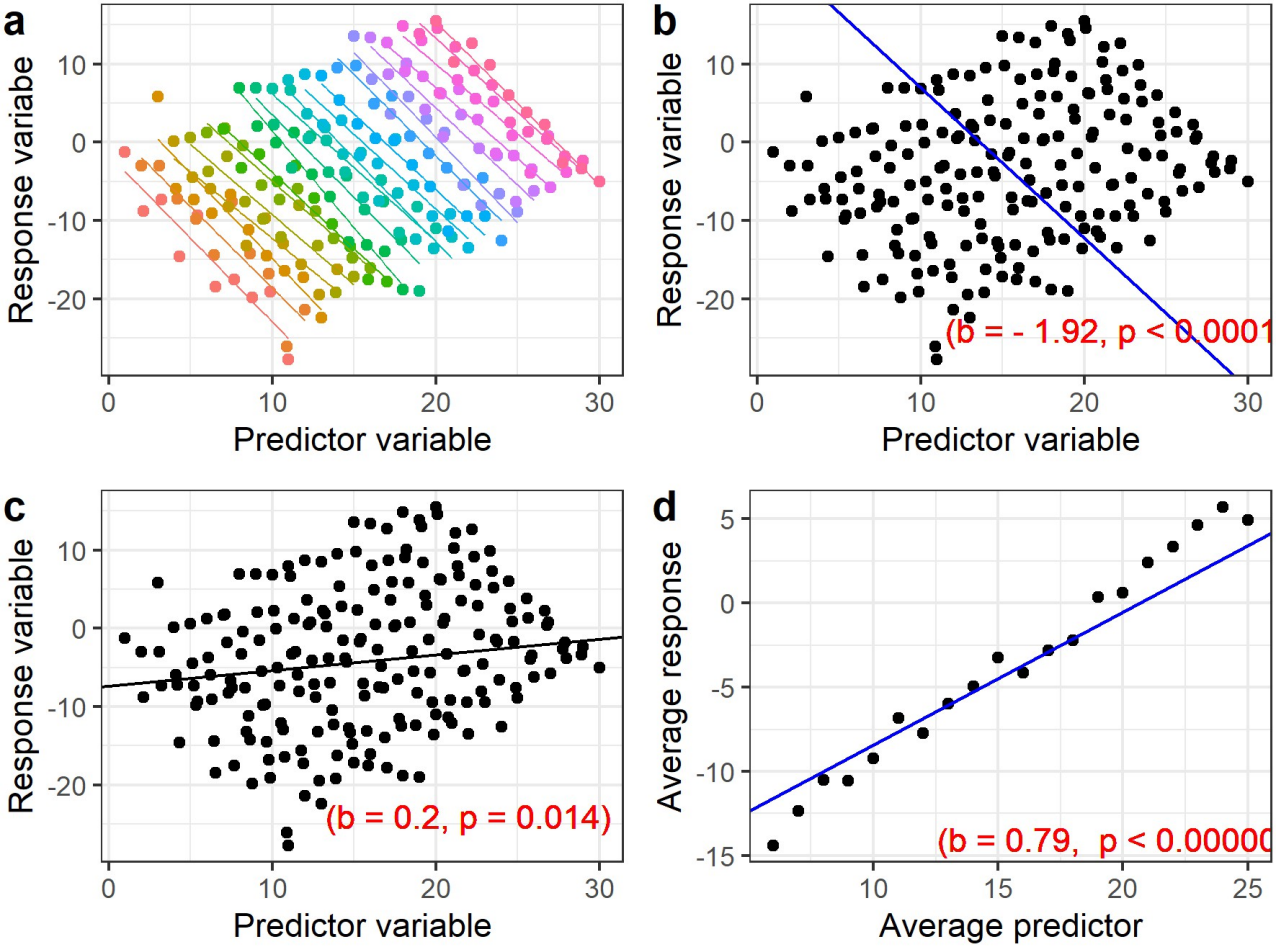
Analyses ignoring and taking into account repeated measures. a) The actual data from 20 individuals (coloured by person), b) Mixed model taking into account subject effects yielding desired results, c) Linear regression model ignoring subject effects leading to inaccurate results with wrong direction of effect and d) Linear regression considering averages per individual giving different direction for the relationship.

Studies using these monitors usually increase the precision of estimating the relationship between glucose and electrocardiographic measurements, thus increasing the power to detect any association [7]. However, data collected on the same individual tend to be similar (yielding correlated or dependent data) and require analyses that take into account that correlation. These data can be analysed in three ways; i) analysing the raw data, using methods that take into account the complex structure of the data (Fig 1b), ii) ignoring the repeated nature of the data and treating all data as independent (Fig 1c), iii) calculating summary statistics per individual and use these aggregate measures in the primary analysis (Fig 1d).

The advantage of (i), i.e. summary statistics, is that the techniques and results are easily understood in the absence of advanced statistical knowledge [7] and they resolve the issue of dependency in the data [8]. However, temporal trends cannot be established from aggregate data and an enormous information loss occurs in calculating these summaries [9]. This will lead to p-values which are too high, confidence intervals which are too wide, and a high false negative rate. For example an individual wearing a CGM for 24 hours provides 288 measurements of glucose (if measured every 5 minutes), but using aggregate data only provides one summary data point for example, glucose variability measure such as standard deviation. This loss of information results in a loss of power to detect experimental effects [10]. Fig 1d shows the results by calculating averages for both the response and predictor which even give different direction of effect (slope = 0.79, p-value <0.00001).

The issue with (ii), i.e. analysing the data as independent, is that it may produce misleading results if there are meaningful individual differences, as illustrated by Bakdash et al. [8]. The results will have p-values which are too small, confidence intervals which are too narrow, and produce many false positive findings. This will in turn waste future research efforts to replicate associations which are not true. Failure to take into account the dependency may even give an effect in the opposite direction (slope = 0.02, p-value0.014) as shown in Fig 1c.

Approach (iii) including mixed models [11] and functional data analysis (FDA) [12] take into account the individual correlation in the data hence giving more reliable results (slope = -1.92, p-value<0.00001) as illustrated by Fig 1b. From a simple dataset, it can be clearly seen that the methods can pick the true direction of the effect compared to the other two methods. These methods lead to results with more statistical power and minimize type I error rate [10]. However, the methods may need advanced statistical skills to be applied properly and understood.

In this systematic review, we aim to examine the methods of data collection and statistical analyses of identified studies by outlining (a) the settings and objectives of studies; (b) the technologies used for data collection and (c) the methods utilised for data analysis. In particular, we assessed whether the proposed analyses included careful consideration of the statistical complexities arising from nonlinear patterns and both between and within-subject variability.

### Research questions

- What are the objectives of studies collecting simultaneous CGM and ECG data?
- Do methods used in analysing CGM and continuous ECG data fully optimise the data collected?

## Materials and methods

### Eligibility criteria

Studies were included if they met all the following four inclusion criteria: 1) both CGM and continuous ECG were measured simultaneously on human participants; 2) the methods used and analysis results were described; 3) inclusion of participants with either type 1 diabetes mellitus (T1DM), type 2 diabetes mellitus (T2DM) or a mixture of T1DM and T2DM; and 4) studies were reported in English. Studies were excluded if: 1) only of CGM or continuous ECG was used, instead of both, 2) ECG and CGM data were not obtained simultaneously; 3) CGM and continuous ECG were used in participants without diabetes or in animals.

### Information sources

Relevant studies were searched using the PubMed and Web of Science platforms from inception of their databases up to 31^st^ May 2021. The search terms used for both databases are shown in S1 Table. Additional studies were searched using reference tracking. Reference lists of included articles were searched for additional citations.

### Selection process

Initial search and checking were performed by BC according to the inclusion and exclusion criteria, subsequently checked independently by AJS. Reporting was in accordance with the Preferred Reporting Items for Systematic Reviews and Meta-Analyses (PRISMA) guidelines [13] as shown in S2 Table. No protocol was published for this research.

### Data collection process

From each article, we extracted the study objectives, design, population, sample size, age and gender profile of participants, duration, variables collected, outcomes, statistical methods and technology used to collect data. Data were extracted by BC and verified by AJS.

### Data items

The outcomes of interest for this review are:

1. the study objectives,
2. technologies used and
3. statistical methods used.

### Study risk of bias assessment

Risk of bias was performed based on the Cochrane Collaboration’s tool [14] for assessing risk of bias in randomised trials as shown in S3 Table. In addition, a critical appraisal was also done using the Joanna Briggs Institute (JBI) Critical Appraisal Checklist [15] for analytical cross-sectional studies.

### Certainty of evidence

Certainty of evidence was performed following Grading of Recommendations, Assessment, Development and Evaluations (GRADE) domains (16).

### Synthesis methods

A post-hoc meta-analysis would have been performed, if three or more studies provided any relevant meta-analysable data or outcome (e.g. a regression of heart rate on glucose), and if appropriately analysed. A list of available results from the studies is created otherwise. Measures used and their estimates and confidence intervals were recorded.

### Patient and public involvement

No patients were involved in this study.

## Results

**Fig 2:**
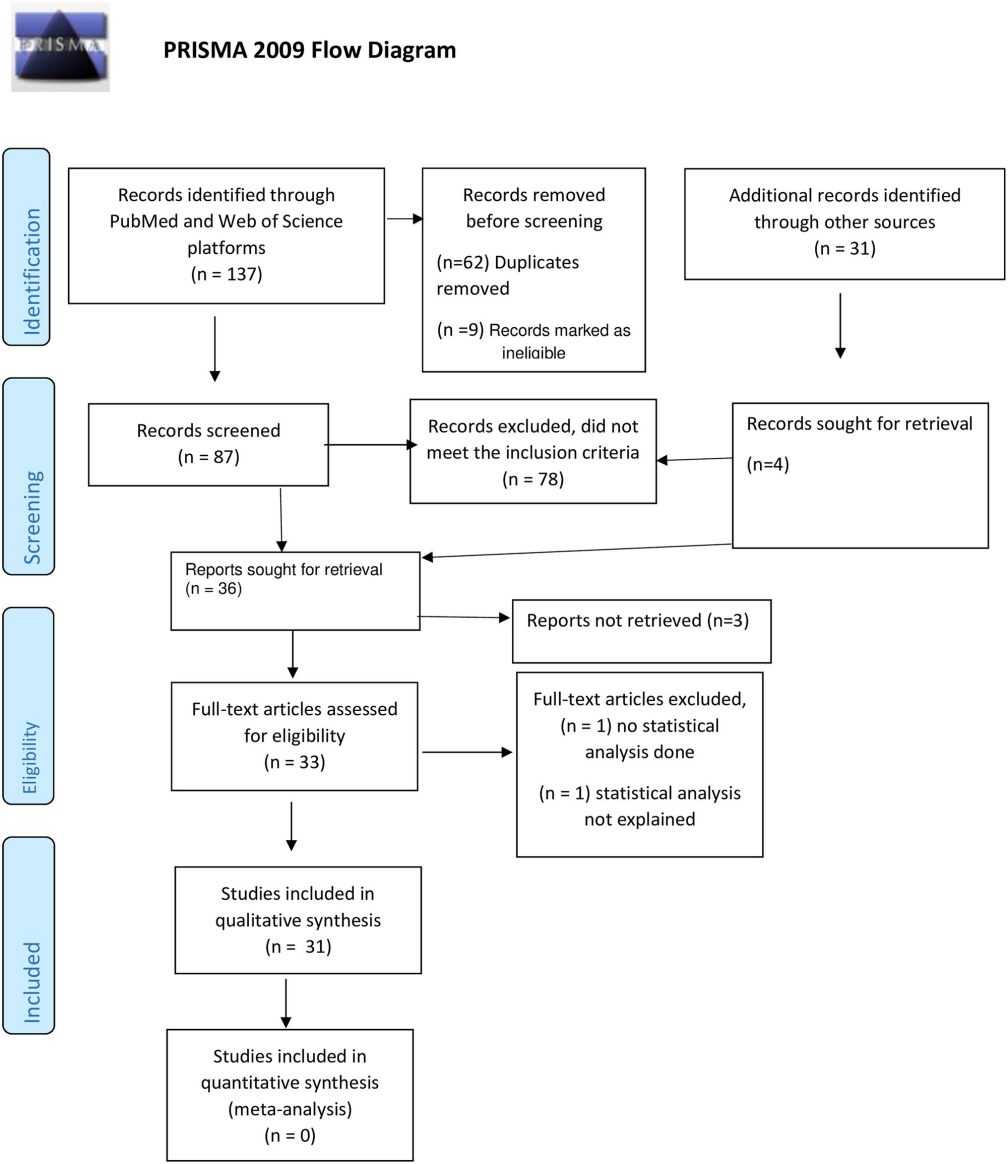
PRISMA flow chart [13].

**Fig 3:**
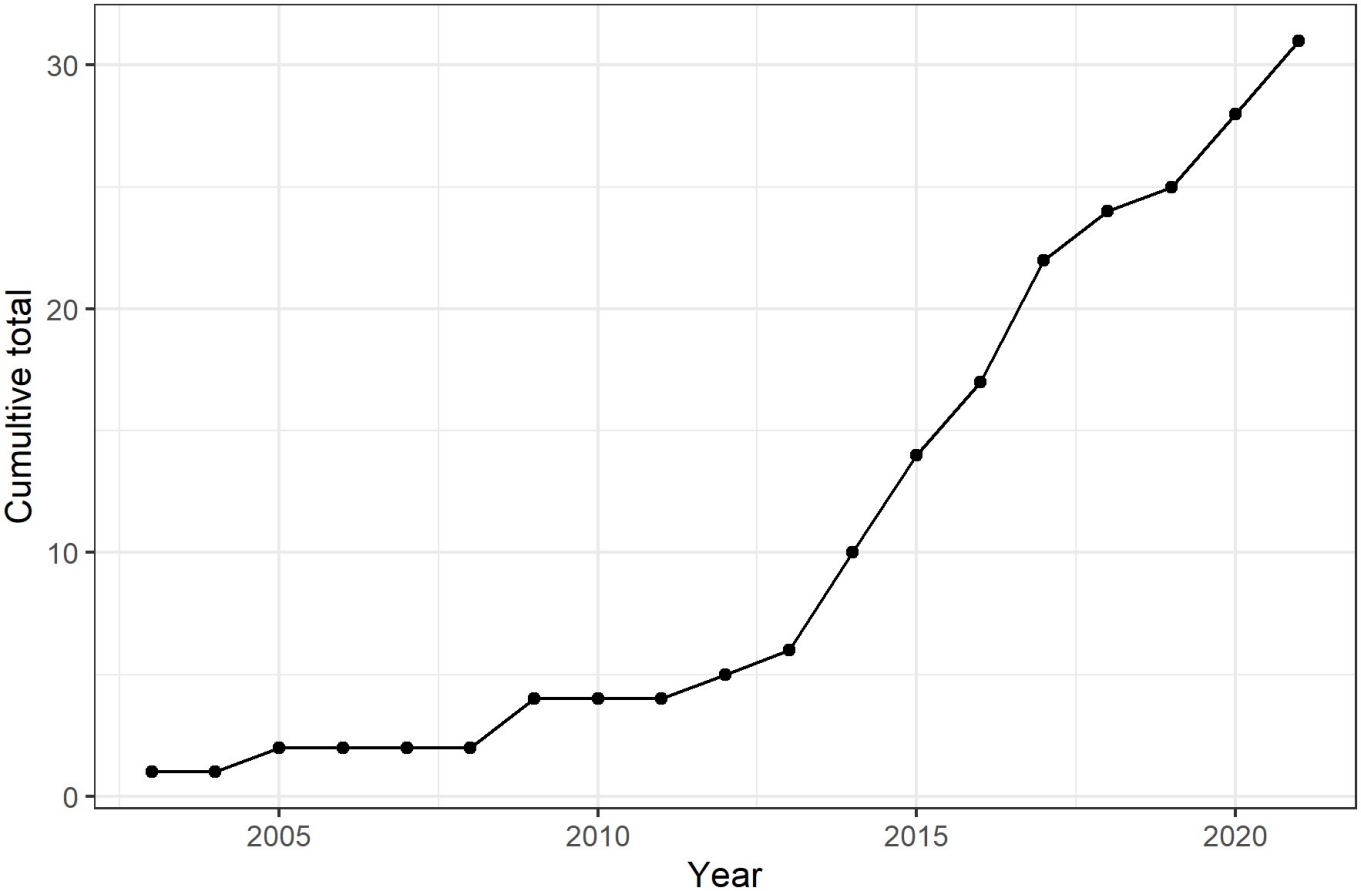
Cumulative number of publications by year.

From the literature search and filtering based on the inclusion criteria, we identified 31 articles published from 2003 to 2021. Fig 2 shows a PRISMA flow chart of the screening and selection results for this review. Fig 3 shows the cumulative number of articles published over time. A qualitative analysis was performed using 28 studies (i.e. some studies published multiple papers on the same data) with a total of 1099 participants, as summarised in S5 Table.

### Study risk of bias assessment

Study risk of bias assessment is shown in S4 Table. Overall, risk of bias was low across included studies. Almost all studies clearly defined the inclusion and exclusion and some randomised trials were blinded. In addition, the data collection tools minimise occurrence of missing data which may happen due to device failure.

### Certainty of evidence

Precision and consistency were low as the studies report different outcomes and hence incomparable confidence intervals. Indirectness was low as the majority of the study participants represent the intended population of interest, that is, diabetic patients. Publication bias is high as majority of the studies aggregate the data and perform simplified analysis which may lead to either false positive or false negative findings.

### Study population, design and objectives

Twelve studies focused on T1DM, 13 on T2DM and three studies included patients with either T1DM or T2DM. Sample sizes ranged from 10 to 102. Duration of CGM and ECG monitoring ranged from 20 hours to 10 days. Participant’s age ranged from 17 to 90 years.

Fourteen studies aimed at finding the effect of hypoglycaemia on heart rate and ECG changes, six evaluated glucose variability (GV) and ECG, three compared CGM and ECG between treatment and control groups, three determined the full relationship between sensor glucose and ECG and evaluated the association between heart rate variability and interstitial glucose variations and two attempted to predict glucose levels from ECG and CGM data.

### Technologies used and variables of interest

Quite a number of technologies were used for both CGM and Holter monitoring. The most commonly used CGM was the Medtronic iPro2 (ten studies), followed by the Dexcom G4 and FreeStyle Navigator II (four studies each), then GlucoDay, guardian real time CGMS and the MMT-7002 sensor (three studies each). Other CGM systems used were the Medtronic Minimed Gold and an unspecified Medtronic Minimed which were used in two studies each. Each study used a different ECG monitor except those done by same investigators [16, 17].

All studies collected sensor glucose from the CGM which they aggregated according to their research question. For example, those interested in GV, calculated GV measures such as the standard deviation, and those interested in hypoglycaemia recorded the count and duration hypoglycaemic episodes. Commonly recorded ECG variables were the heart rate, QT, RR, and QRS intervals, which were then aggregated for analysis, e.g. by calculating heart rate variability for each participant in order to estimate cardiac autonomic activity [18].

### Statistical analysis

Of the 31 articles included in this systematic review, 23(74%) studies calculated summary measures from the data, three (10%) ignored dependency and 7(23%) correctly analysed their data. Seven studies (23%) considered the repeated nature of the data and fitted hierarchical models. Of these, five did not perform simple analysis (ignoring dependency) for the variables which they applied the advanced analysis (taking into account individual correlation). Kubiak et al. [19] applied both simple and advanced analysis and found no association without controlling for individual correlation and a significant relationship between QT corrected for heart rate (QTc) and glucose after controlling for subject effects. We also performed both simple and advanced and both approaches gave similar conclusions [20]. One study (3%) FDA taking into account the trajectory of the data over time. It should be noted that, of the seven articles who took into account the repeated nature of the data, four articles have overlapping authors, leaving four distinct articles that used advanced analyses.

The quantitative results of included studies are presented in S6 Table. Unfortunately, due to the diversity of variables, study designs and statistical methods, no post-hoc meta-analysis of these results is possible. Moreover, most statistical analyses methods employed underutilised the information collected and used methods that may give appropriate results hence discouraging the pooling of results.

## Discussion

We performed a systematic review and critically appraised 31 articles (28 studies) which collected ECG and glucose continuously over time extracting information about study objectives, technologies used to collect data and statistical analysis performed. It was found that the most common analysis of such data is calculating summary metrics per individual and applying simplified analysis, others performed analysis ignoring data dependency and some used methods controlling for the dependency. The most commonly used CGM was the Minimed iPro2 and we found no study that has looked at which of the CGM systems is most suitable and why. The main objective for many studies was to determine the relationship hypoglycaemia and ECG changes.

Seven studies have applied analyses that take into account the individual correlation. Of these, two analysed data by employing both simplified analyses (summary measures or treating data as independent) and advanced analysis (mixed models and FDA). One study found different results from the two methods, that is, ignoring dependency resulted in no association between QTc and glucose whilst controlling for correlation yielded a significant relationship [19]. We found similar results using both simplified and using the raw data [20]. Our study finds that QTc increases with an increase in glucose, which contradicted other studies that used summarised data. These results have shown that in some cases, the results may or may not be different between the simple analysis and the one accounting for dependency in the data, but the magnitude of the effect will be different. It is therefore important to do both analyses.

We saw huge uptake in simplified statistical analysis which may be due to their simplicity in interpretation compared to the advanced analysis which model all individual data. In addition, some summary measures calculated for example, heartrate variability show the stability of the heart better compared to the raw data. Therefore, depending on the objective of the study, some metrics can be useful for interpretation, however other metrics such as area under a curve are simple but not easy to interpret.

The previous barriers to the widespread implementation of CGM include issues with accuracy and user friendliness which have been largely resolved with newer and more advanced technology [21]. Furthermore, better accessibility and increased advocacy for people with diabetes also resulted in the increased uptake of CGM [22]. This is also mirrored by the easier access to continuous cardiac monitoring.

Many systematic reviews have been published in cardiology and diabetes. Most investigated the relationship between biomarkers and risk factors between diabetes and cardiology. For example, Selvin et al. 2004 [23] performed a meta-analysis of observational studies on the association between glycosylated hemoglobin and cardiovascular disease in diabetics. Zelniker et al. 2019 [24] performed a systematic review and meta-analysis on cardiovascular outcome trials of SGLT2 in patients with type II diabetes. In terms of statistical methods in diabetes and cardiology, Kavakiotis et al. [25] and others [26, 27] looked at data mining and statistical methods used in diabetes mellitus diagnosis in general, without a link to ECG data. This study looked at modelling using regression models for CGM and ECG data. The strength of this systematic review is that it is the first to examine the methods of data collection, technology used and statistical analyses in studies combining simultaneous CGM with ECG monitoring in people with diabetes. It allows us to examine the transparency and the adequacy of the analyses in these studies and inform future recommendations. The limitation of this systematic review is that we were unable to perform a formal meta-analysis of the included studies due to their diversity in design and statistical methods used since some of the methods applied may not yield desired results. However, this did not alter the overall conclusion of our systematic review.

We recommend the use of statistical methods that can use the data with as little aggregating as possible in order to prevent loss of important information from the data. The methods should control for the within and between subject variability. The analysis of such data should take into account the individual changes over time. For such data we recommend summary measures and simple analysis as exploratory analysis and methods such as mixed models [9, 28] and generalised estimating equations (GEE) [28] to take into account the data dependency whenever the subjects have repeated measurements. We further recommend the use of functional data analysis [12, 29] that is more flexible to the trajectory of the data. These approaches will lead to increased statistical power and better evidence in this area of growing interest.

## Supporting information

S1 Table Search strategy. (DOCX)

S2 Table PRISMA 2020 checklist. (DOCX)

S3 Table (XLSX)

S4 Table Joanna Briggs Institute (JBI) critical appraisal checklist. (DOCX)

S5 Table Study characteristics of the included studies. (DOCX)

S6 Table Quantitative findings from included studies. (DOCX)
