# Supplementary material for: Statistical techniques used in analysing simultaneous continuous glucose monitoring and ambulatory electrocardiography in patients with diabetes: A systematic review
Source: PLoS One. 2023 Feb 24;18(2):e0269968. doi: 10.1371/journal.pone.0269968 (PMC9955667; doi:10.1371/journal.pone.0269968)
Supplement: S1 Table — (DOCX) [file pone.0269968.s001.docx]

S1 Table. Search strategy

| **Database** | **Search terms** |
| --- | --- |
| PubMed | #1 diabetes[All Fields] OR diabetic[All Fields] OR diabetes mellitus[MeSH Terms]) OR diabetes mellitus[All Fields]  #2 “Continuous glucose monitoring” [ All Fields] OR “Continuous glucose monitor” [ All Fields]) OR CGM[All Fields]  #3 ((((((“continuous electrocardiogram”[ All Fields]) OR  (“continuous electrocardiography”[ All Fields])) OR “  (“Holter monitoring”[All Fields])) OR ("electrocardiography, ambulatory"[MeSH Terms]) OR  (“Holter electrocardiography”[ All Fields])) OR (Holter[All Fields]))  OR(“Holter monitor”[ All Fields])) OR  (“Holter electrocardiogram”[ All Fields])  #4 #1 AND #2 AND #3 |
| Web of Science | ALL=( diabetes OR diabetic) AND ALL=( Continuous glucose monitoring OR Continuous glucose monitor OR CGM) AND ALL=( Continuous Electrocardiogram OR Ambulatory Electrocardiogram OR Holter monitoring OR Ambulatory Electrocardiography OR Ambulatory Electrocardiogram OR Holter OR Continuous Electrocardiography ) |
