# Supplementary material for: Statistical techniques used in analysing simultaneous continuous glucose monitoring and ambulatory electrocardiography in patients with diabetes: A systematic review
Source: PLoS One. 2023 Feb 24;18(2):e0269968. doi: 10.1371/journal.pone.0269968 (PMC9955667; doi:10.1371/journal.pone.0269968)
Supplement: S4 Table — (DOCX) [file pone.0269968.s004.docx]

S4 Table. Joanna Briggs Institute (JBI) Critical Appraisal Checklist

| Author | Were the criteria for inclusion in the sample clearly defined? | Were the study subjects and the setting described in detail? | Was the exposure measured in a valid and reliable way? | Were objective, standard criteria used for measurement of the condition? | Were confounding factors identified? | Were strategies to deal with confounding factors stated? | Were the outcomes measured in a valid and reliable way? | Was appropriate statistical analysis used? | Overall appraisal: |
| --- | --- | --- | --- | --- | --- | --- | --- | --- | --- |
| Eguchi, K et al. | Yes | Yes | Yes | Yes | No | No | Yes | Yes | Include |
| Mezquita-Raya P et al. | Yes | Yes | Yes | Yes | Unclear | Unclear | Yes | Yes | Include |
| Matsushita Y et al. | Yes | Yes | Yes | Yes | Unclear | Unclear | Yes | Yes | Include |
| Novodvorsky P et al. | Yes | Yes | Yes | Yes | No | No | Yes | Yes | Include |
| Middleton TL et al. | Yes | Yes | Yes | Yes | No | No | Yes | Yes | Include |
| Kubiak T et al. | Yes | Yes | Yes | Yes | No | Unclear | Yes | Yes | Include |
| Cypryk K et al. | Yes | Yes | Yes | Yes | No | Unclear | Yes | Yes | Include |
| Gill G V et al | Yes | Yes | Yes | Yes | No | No | Yes | Yes | Include |
| Koivikko ML, et al | Yes | Yes | Yes | Yes | No | No | Yes | Yes | Include |
| Koivikko ML, et al | Yes | Yes | Yes | Yes | No | No | Yes | Yes | Include |
| Pistrosch F, et al. | Yes | Yes | Yes | Yes | No | No | Yes | Yes | Include |
| Shimabukuro M, et al. | Yes | Yes | Yes | Yes | No | No | Yes | Yes | Include |
| Desouza C et al | Yes | Yes | Yes | Yes | No | No | Yes | Yes | Include |
| Probstfield JL et al. | Yes | Yes | Yes | Yes | Unclear | Unclear | Yes | Yes | Include |
| Probstfield JL et al. | Yes | Yes | Yes | Yes | Unclear | Unclear | Yes | Yes | Include |
| Lee AS et al. | Yes | Yes | Yes | Yes | No | No | Yes | Yes | Include |
| Klimontov V et al. | Yes | Yes | Yes | Yes | No | No | Yes | Yes | Include |
| Chow E et al. | Yes | Yes | Yes | Yes | Unclear | Unclear | Yes | Yes | Include |
| Ali Abdelhamid Y et al. | Yes | Yes | Yes | Yes | Unclear | Unclear | Yes | Yes | Include |
| Abobarin-Adeagbo A et al. | Yes | Yes | Yes | Yes | No | No | Yes | Yes | Include |
| Yang D et al. | Yes | Yes | yy | Yes | No | No | Yes | Yes | Include |
| Pertseva NO et al. | Yes | Yes | Yes | Yes | No | No | Yes | Yes | Include |
| Cichosz SL et al. | Yes | Yes | Yes | Yes | Unclear | Unclear | Yes | Yes | Include |
| Stahn A et al. | Yes | Yes | Yes | Yes | No | No | Yes | Yes | Include |
| Borgognoni L et al. | Yes | Yes | Yes | Yes | No | No | Yes | Yes | Include |
| Kalopita S et al. | Yes | Yes | Yes | Yes | No | No | Yes | Yes | Include |
| Cichosz SL et al. | Yes | Yes | Yes | Yes | Unclear | Unclear | Yes | Yes | Include |
| Cichosz SL et al. | Yes | Yes | Yes | Yes | Unclear | Unclear | Yes | Yes | Include |
| Richardson T et al. | Yes | Yes | Yes | Yes | No | No | Yes | Yes | Include |
| Bernjak A et al. | Yes | Yes | Yes | Yes | Unclear | Unclear | Yes | Yes | Include |
| Charamba B et al. | Yes | Yes | Yes | Yes | Unclear | Unclear | Yes | Yes | Include |
