# Supplementary material for: Statistical techniques used in analysing simultaneous continuous glucose monitoring and ambulatory electrocardiography in patients with diabetes: A systematic review
Source: PLoS One. 2023 Feb 24;18(2):e0269968. doi: 10.1371/journal.pone.0269968 (PMC9955667; doi:10.1371/journal.pone.0269968)
Supplement: S5 Table — (DOCX) [file pone.0269968.s005.docx]

S5 Table. Study characteristics of the included studies.

| **Author** | **Study objective** | **Population** | **Sample size** | **ECG Variables** | **Age range** | **Duration and Frequency** | **Study outcome** | **Statistical methods** | **Technology used** |
| --- | --- | --- | --- | --- | --- | --- | --- | --- | --- |
| Eguchi, K., Komori, T., Saito, T., Hoshide, S. & Kario, K | Test the hypothesis that alpha-glucosidase inhibitor (α-GI), miglitol, is effective in protecting the cardiovascular system in type 2 diabetes mellitus (T2DM).^37^ | Heart disease with T2DM | 19(13male) | Period to record arrhythmia, T-wave alternans (TWA), and heart rate  turbulence. RR interval | 61.7 ± 14.7 | 48 hours  CGM(5mins) | Glucose variability,  arrhythmia,  heart rate variability, and T-wave alternans | t-test, ANOVA, chi-square test , Wilcoxon signed rank test, Pearson’s correlation | SEER® Light Holter monitor (GE Healthcare, Tokyo).  The CGMS® System Gold™; Medtronic, Fridley, MN, USA) |
| Mezquita-Raya P, Reyes-García R, de Torres-Sánchez A, Matarín MG, Cepero-García D, Pérez de Isla L. | Evaluate the changes in ECG, evaluated by Holter monitoring, induced by clinical hypoglycaemia  in participants with type 1 diabetes (T1DM) and type 2 diabetes (T2DM)  .^18^ | T1DM and T2DM with high cardiovascular risk | 26(16male) | Heartrate, QRS interval, QTc interval, PR interval | >50 | 61.7 ± 18.4 hours | QTc interval, hypoglycaemic episodes | Fisher‘s exact, U Mann-Whitney or t-Student tests, mean and standard deviation | Five-lead Holter monitoring (BL-900 Braemar, Inc, Norav Medical  NH301-2.4.5 software),  CGM, (Dexcom G4-Platinum, range 40–400 mg/dl) |
| Matsushita Y, Takata Y, Kawamura R, Takakado M, Hadate T, Osawa H. | Investigate the relation between autonomic nerve activity (ANA) around  wake-up and either morning or daily GV.^27^ | T2DM | 41 (20male) |  |  | 24hours  (5mins) | Glucose variability, Heart rate variability | Multiple regression | iPro2 (Medtronic MiniMed, Northridge, CA, USA), spectral analysis (Mem Calc system; Nihon-Kohden, Japan) |
| Novodvorsky P, Bernjak A, Chow E, Iqbal A, Sellors L, Williams S, Fawdry RA, Parekh B, Jacques RM, Marques JLB, Sheridan PJ, Heller SR. | Examine the effect of nocturnal and daytime clinical hypoglycaemia on electrocardiogram  (ECG) in young people with type 1 diabetes.^19^ | T1DM | 37(19male) | QT interval. RR interval | <=50 | 96hours  CGM(10mins) ECG(5mins) | Hourly mean IG, Hourly counts for each type of arrhythmia, Heart rate variability, QTc, hypoglycaemic event | GEE, negative binomial model ,Mann-Whitney U test, t-test | Ambulatory ECGs (H12+; Mortara  Instrument, Milwaukee, WI), CGM (FreeStyle Navigator II; Abbott  Diabetes Care, Maidenhead, U.K.). |
| Middleton TL, Wong J, Molyneaux L, Brooks BA, Yue DK, Twigg SM, Wu T. | To determine the effect of sulfonylurea-related hypoglycaemia on cardiac repolarization and ectopy in the setting of well-controlled type 2 diabetes.^20^ | T2DM receiving  treatment with sulfonylurea therapy. | 30(18male) | QT, QTc, RR |  | 48hours  (5mins) | Change in QTc  length, QTc dynamicity, QTc prolongation | Fisher’s exact test, t-test, chi-square test | GE Seerlight Extend Compact  Digital Holter (GE Medical Systems, Milwaukee, WI). iPro2 – Medtronic USA |
| Kubiak T, Wittig A, Koll C, Mraz B, Gustav J, Herrmann U, Weber H, Kerner W | Derive parameters for  direction and strength of the glucose–QTc association.^12^ | T1DM | 20(10male) | QTc | 22–65 | 48hours | QTc, sensor glucose | Regression modelling, Hierarchical (mixed) regression modelling | GlucoDay (Menarini Diagnostics, Florence, Italy). two-channel-lead Spacelabs Del Mar Reynolds Lifecard_ system (Spacelabs Healthcare, Issaquah, WA). |
| Cypryk K, Bartyzel L, Zurawska-Klis M, Mlynarski W, Szadkowska A, Wilczynski J, Nowakowska D, Wozniak LA, Fendler W. | Investigate association between maternal glycaemic fluctuations  and foetal heart rate variability.^21^ | T1DM | 14 females | Foetal heart rate | 25-37 | >20hours (5mins) | FHR  Accelerations (large/small) | Generalized mixed-effect logistic regression | Monica AN24_ device (Monica Healthcare Ltd., Nottingham, United Kingdom).  iPro_2; Medtronic,  Minneapolis, MN) |
| Gill G V., Woodward A, Casson IF, Weston PJ | Investigate ECG QT  prolongation with subsequent ventricular tachyarrhythmia  in response to nocturnal hypoglycaemia.(Gill et al. 2009) | T1DM | 25(12males) | QTc | 20-50 | 2 x 24hours  And meter glucose | QTc | t-test. Mean and standard deviation | (Pathfinder 700 Issue 7.203 continuous QTc analyser; Del Mar  Reynolds, Hertford, UK). MMT-7002  sensors (both supplied by Medtronic MiniMed, Minneapolis,  MN, USA). |
| Koivikko ML, Kenttä T, Salmela PI, Huikuri H V., Perkiömäki JS | Evaluate the influence of spontaneous nocturnal hypoglycaemia  on repolarisation morphology in a ‘real life’situation.^23^ | T1DM | 11(4males) | QT, QRS,  T-wave  Heartrate | 19-41 | 3 x overnight | Heart rate, QT interval, QTD, QTc, QT_NC_, dispersion, T-wave morphology | Paired t-test, Wilcoxon test | MMT-7002 sensor (Medtronic Diabetes, Northridge, California, USA). the RAFE lead system |
| Koivikko ML, Tulppo MP, Kiviniemi AM, Kallio MA, Perkiömäki JS, Salmela PI, Airaksinen KEJ, Huikuri H V | To assess the effect of spontaneous hypoglycaemia on heartrate variability during sleep.^25^ | T1DM | 37(15male) | RR interval | 19-41 | 3x overnight | Heart rate variability, RR interval variability | Paired t-test, Wilcoxon test | MMT-7002 sensor (Medtronic Diabetes, Northridge, California, USA). Two dermal electrodes with a real time microprocessor based QRS detection (Polar Electro, Kempele Finland). |
| Pistrosch F, Ganz X, Bornstein SR, Birkenfeld AL, Henkel E, Hanefeld M | Determine whether there is a relationship between hypoglycaemic events (HE) and severe cardiac arrhythmias in participants with type 2 diabetes and established clinical risk factors under real-world conditions.^24^ | T2DM and documented cardiovascular disease, | 94(71 male) | Heart rate, QTc time,  couplets, triplets and ventricular tachycardia (VT). | 50-80 | 5days | Ventricular arrhythmias, hypoglycaemic events amplitude of R–R intervals,  Heart rate variability SD of normal R–R intervals (SDNN). | T-test, Man Witney test, Chi-square, multiple regression analysis | CGM:(Medtronic ipro2_). ECG: pro_ system (Am edtec). |
| Shimabukuro M, et al. | To evaluate the short-term effects of glucose fluctuations on HRV  and sympathetic nervous system (SNS) activity in adults with T2DM and recent ACS. Examine the effect of suppressing  glucose fluctuations with miglitol on these variables.^38^ | T2DM with recent acute coronary syndrome | 39(31 male) | RR interval |  | 24hours | Heart rate variability, Glucose variability | t-test, paired t-test, Wilcoxon test, Fisher’s exact test | (Medtronic iPro2) |
| Desouza C, Salazar H, Cheong B, Murgo J, Fonseca V | To determine whether episodes of hypoglycaemia were more likely to be associated with cardiac ischemia than normoglycaemia or hyperglycaemia. ^40^ | T2DM with coronary artery disease | 19(12male) | ST segment |  | 72 hours | Hypoglycaemic episode, and ischemic episodes | Yates corrected  Chi-square test. | GE/  Marquette Holter, CGM (Minimed, CA) |
| Probstfield JL et al. | Test whether a glucagon-like peptide 1  agonist-based regimen can reduce GV and cardiometabolic risk markers while  maintaining similar A1C levels in people with insulin-requiring type 2 diabetes and high cardiovascular risk.^14^ | T2DM and high cardiovascular risk. | 102(65male) |  | 40-75 | 7-10days | Glucose variability  measures, critical arrhythmias and significant abnormalities | Two sample  t test, an ANCOVA model, Wilcoxon rank sum test, logistic  regression | CGM (Dexcom  Seven Plus or Gen 4),  Holter  (Medicomp) |
| Probstfield JL et al. | Test the hypothesis: Glycaemic variability contribute to adverse medical outcomes of type 2 diabetes.^15^ | T2DM and high cardiovascular risk. | 102 (65male) | QTc, Heartrate |  | 7-10 days x3 | Change in the coefficient of variation  (CV), glucose variability measures,  . panic and alert rhythms. (expressed as percent  of beats per day or number of beats) | Two sample  t test, an ANCOVA model, Wilcoxon rank sum test. | (SMBG) using the Bayer Contour meter,  CGM (Dexcom  SEVEN PLUS, ECG monitoring (Medicomp Holter) |
| Lee AS, Brooks BA, Simmons L, Kilborn MJ, Wong J, Twigg SM, Yue DK | Explore the effect of ‘spontaneous’ hypoglycaemia and hyperglycaemia on QT interval during free living conditions.^60^ | T1DM and T2DM | 14 | QT interval  Heatrate | 62.0 7.2 | 48hours, every 5 minutes | QTc, periods of hypoglycaemia, euglycaemia, and hyperglycaemia. | Paired t-test | CGM (Medtronic iPro2)  Holter: MARS V7.2 system |
| Klimontov V V, Myakina NE, Tyan N V | Investigate the associations of frequency-  domain HRV parameters with current and antecedent  interstitial glucose fluctuations in insulin-treated  type 2 diabetic patients at high cardiovascular risk.^41^ | T2DM women | 67 women | Heartrate | 48-78 | >48 hours CGM  24 hours ECG | mean glucose and 8 intraday GV  indices, including standard deviation (SD), 2-h continuous  overlapping net glycemic action (CONGA2), lability  index, J-index, mean amplitude of glucose excursions  (MAGE), mean absolute glucose (MAG), low blood glucose  index (LBGI) and high blood glucose index (HBGI), HRV parameters, Cardiovascular autonomic neuropathy | Multiple stepwise regression, ANOVA Kruskal-Wallis, Mann–Whitney test or Wilcoxon matched-pair test | CGM Medtronic MiniMed iPro2 |
| Chow E, Bernjak A, Williams S, Fawdry RA, Hibbert S, Freeman J, Sheridan PJ, Heller SR. | Examine the frequency of  arrhythmias during spontaneous hypoglycemia and hyperglycemia  versus euglycemia in patients with type 2  diabetes and cardiovascular risk. The effect of glucose on  cardiac autonomic tone and repolarization.^42^ | T2DM with  cardiovascular risk. | 25 (13male) | atrial ectopic beats, bradycardia, VPBs,  and complex VPB, R-R intervals, QT intervals, Heartrate | 61-71 | 5 days | hypoglycemic episode, hyperglycemic episode, euglycemic period, HRV, QT intervals | Summary statistics, paired t test, independent t test, Mann-Whitney U test, or Fisher exact.GEE, Poisson model | FreeStyle Navigator  Continuous Glucose Monitoring System; Abbott  Diabetes Care, Maidenhead, U.K.).  ECGs (Lifecard 12; Spacelabs Healthcare, Hertford,  U.K.) |
| Ali Abdelhamid Y, Bernjak A, Phillips LK, Summers MJ, Weinel LM, Lange K, Chow E, Kar P, Horowitz M, Heller S | The hypotheses were that hypoglycemia occurs  frequently, may be asymptomatic, and is associated  with arrhythmias and abnormalities of cardiac repolarization  and autonomic tone.^34^ | T2DM ICU survivors | 30(19 male) | QT interval, T-peak to T-end, R-R interval, | 65 (13) | 5 days, every 5 minutes | Counts of arrhythmia, HRV, proportion of participants with hypoglycemic episode, time in HE,  proportion of asymptomatic hypoglycemic episodes; duration and timing of hypoglycemic episodes; hypoglycemia  awareness; proportion of participants experiencing  a hyperglycemic episode; proportion of participants  with cardiovascular autonomic neuropathy; | Summary statistis, Student’s *t*, Mann-Whitney, or  Fisher exact tests. GEE | CGM: Dexcom  G4 Platinum monitor  Holter: H12+;  Welch Allyn Australia |
| Abobarin-Adeagbo A, Wienke A, Girndt M, Pliquett RU | Test hypothesis that consecutively hospitalized insulin-treated diabetes patients with hypertensive crisis on admission have a propensity for hypoglycemic episodes in post-admission continuous glucose monitoring (CGM)^36^ | T1DM and T2DM, T3c DM | 53(22 male) | RR intervals | 18–99 | 14 days | hypoglycemic episodes , HRV, HbA1c, BMI. eGFR | Summary statistics, ANOVA | CGM: FreeStyle libre, Abbott Diabetes Care, Abbott GmbH |
| Yang D et al. | To comprehensively assess the effects of metformin added to insulin on metabolic control, insulin sensitivity, and cardiovascular autonomic function in adolescents with type 1 diabetes^35^ | T1DM | 17(10 male) | RR intervals | 12-18 | 3xCGM 72hours  3xHolter: 24 hours | BMI, BP, change in HbA1c from baseline, GV (MAGE, CV, MODD) and HRV(standard deviations of RR 24 hr , sd of RR 5 min, % interval differences of successive RR intervals greater then 50ms) | Summary statistics, t-test, chi-squared test or Fisher’s exact test, or Mann–Whitney U test | CGM: iPro2 digital recorder, Medtronic DiabetesS  ECG: Marquette, USA |
| Pertseva NO, Gurzhiy O V, Moshenets KI. | Evaluate  the effect of major indicators of control and course of  type 1 diabetes on heart rate variability^31^ | T1DM | 62(25male) | frequency bands in ms^2^  ,total power of the spectrum | 18-45 | 24hours | HRV variables: diurnal  SDNN,  , RMSSD day, pNN50%,  TP, VLF  , LF, HF,  LF/HF,  minimum and maximum  glycemia, HbA1c, C-peptide, creatinine,  GFR and AU. | Correlations, median and quartiles, canonical correlation coefficient, | CGM: iPro2 system (Medtronic MiniMed, USA).  Holter: SDM23  apparatus ( LLC “IKS-Techno” Ukraine) |
| Cichosz SL, Frystyk J, Tarnow L, Fleischer J | To examine  if changes in HRV during hypoglycemia are confounded by the presence of cardiovascular autonomic neuropathy (CAN).^32^ | T1DM | 21(13 men) | frequency bands in ms^2^ | 58(10) | 72 hours | HRV variables SDNN, RMSSD, HF, and TP | paired-samples t-test., linear regression. | CGM: Medtronic MiniMed, Northridge, CA)  Holter : (SpiderView Plus; ELA Medical, Montrouge,  France). |
| Stahn A, Pistrosch F, Ganz X, Teige M, Koehler C, Bornstein S, Hanefeld M | To evaluate the risk of critical arrhythmias related to glycemic variability^29^ | T2DM with CVD | 30 | Heartrate, QT interval | 50-80 | 5 days(every 5 minutes) | average IG and SD; mean amplitude of glucose excursions (MAGE); area under the curve (AUC); minimum and maximum IG; frequency and time of HE, heart rate QTc time; ventricular extrasystoles (VESs); couplets; triplets; and ventricular tachycardias (VTs). HRV (SDNNs) | Summary statistics, correlations, t-test | CGM: Medtronic MiniMed Gold  ECG: Amedtec ECGpro system |
| Borgognoni L, Picciarella A, Stefano A Di, Fontana V, Russo A, Pascucci M, Paris A, Tubani L, Fiorentini A. | Demonstrate  the presence of significant alterations in cardiac  autonomic activity in patients with recent onset type 2  diabetes.^61^ | T2DM | 10(6 male) | RR interval | 58.7 (16.9) | 24hours | HRV(LF,HF, SDNN)  GV | Summary statistics, t-test | CGM: Gluco-Day system (Menarini). |
| Kalopita S, Liatis S, Thomakos P, Vlahodimitris I, Stathi C, Katsilambros N, Tentolouris N, Makrilakis K | Investigate  the relationship between cardiac autonomic function  (evaluated by HRV during continuous ECG recording) and  GV (assessed by simultaneous continuous interstitial tissue  glucose monitoring) in patients with type 2 diabetes^33^. | T2DM | 50 (29 male) |  | 58.4(9.9) | 24hours | HRV(VHF, LF, HF), GV(SDMG, MAGE, 𝑀-value) | Summary statistics, normality tests, Pearson’s correlation | CGM: GlucoDay,  Menarini Diagnostics.  Holter; Spider View  (ELA Medical, France) |
| Cichosz SL, Frystyk J, Tarnow L, Fleischer J | Used developed algorithm (a pattern classification  method) to predict spontaneous hypoglycemia based on CGM and HRV.^44^ | T1DM | 21(13 men) | QRS, Heartrate | 58(10) | 72 hours (every 5 minutes) | HRV(LF, HF, VLF,LF/HF,SDNN, SDANN)  pNNx, RMSSD,  single  measurements of glucose (SMG), | Classification and prediction | CGM; Guardian Real-Time  (Medtronic MiniMed,  Northridge, CA, USA)  Holter: (SpiderView  Plus, ELA Medical, Montrouge, France), |
| Cichosz SL, Frystyk J, Hejlesen OK, Tarnow L, Fleischer J | Investigate whether a novel algorithm that adds information of the complex dynamic/pattern of heart rate variability  (HRV) could improve the accuracy of hypoglycemia as detected by a CGM device.^43^ | T1DM | 10male | RR interval,  Heartrate | 44(10) |  | HRV, sensor glucose | Prediction, and classification, t-test, McNemar test, Normality tests, | CGM Guardian RT; Minimed  Inc, Northridge, CA, USA)  ECG; lead II equipment |
| Richardson T, Thomas P, Ryder J, Kerr D | Examine  the influence of regular ingestion of modest  amounts of caffeine on the frequency  of hypoglycemic episodes and to investigate  the possibility of a relationship between  the augmentation in nocturnal  autonomic function with caffeine and frequency  of nocturnal hypoglycaemia.^62^ | T1DM | 19(9 male) | RR interval | 44.3(9.2) | 2 x 48 hours | HRV, average glucose,  Number, severity, and duration of hypoglycemic episodes | Summary statistics, Normality tests, paired t-test, Spearman’s correlation, Wilcoxon test | CGMS: Medtronic Minimed,  Minneapolis, MN  Holter: miniature  digital recorder (Life Card, Reynolds  Medical). |
| Bernjak A et al. | We examined the effect of spontaneous hyperglycaemia in adults with type 1 diabetes mellitus (T1DM) and without history of cardiovascular disease on heart rate variability (HRV), cardiac repolarisation and incidence of cardiac arrhythmias.^28^ | T1DM | 37(19 male) | RR interval, | 17-50 | 96hours  CGM(10mins) ECG(5mins) | HRV(LF, HF), QT interval and cardiac repolarisation, Hyperglycaemic hours, euglycaemic hours, incidence rate differences, counts of cardiac arrhythmias | linear mixed effects, | CGM: (Freestyle Navigator II, Abbott Diabetes Care, Maidenhead, UK).  Ambulatory ECGs (H12+; Mortara  Instrument, Milwaukee, WI), |
| Charamba B et al. | Investigates the relationship between glucose and QTc (a key cardiovascular measure) using data from continuous electrocardiogram (ECG) and glucose monitors.^17^ | T1DM | 17 male | QT interval | 52.5( 3.8) | 7 days  CGM(5mins) ECG(10mins) | AUC, MADE, QTc interval, IG, | Summary statistics, mixed effects ANOVA, functional regression model | CGMS (iPro^2^, Medtronic, USA)  ECG: (Master Caution device, HealthWatch) |
