# Supplementary material for: Statistical techniques used in analysing simultaneous continuous glucose monitoring and ambulatory electrocardiography in patients with diabetes: A systematic review
Source: PLoS One. 2023 Feb 24;18(2):e0269968. doi: 10.1371/journal.pone.0269968 (PMC9955667; doi:10.1371/journal.pone.0269968)
Supplement: S6 Table — (DOCX) [file pone.0269968.s006.docx]

# S6 Table. Quantitative findings from included studies

| Study | Quantitative Findings | Analysis type |
| --- | --- | --- |
| Mezquita-Raya et al. ^18^ | In participants with hypoglycaemic episodes, mean QTc during hypoglycaemia was longer compared to mean QTc in total (T1D+T2D) group (+4.6 ms, p = 0.037) and T1DM (+5.5 ms, p = 0.048) but not in T2DM (+3 ms, p = 0.459). During hypoglycaemia, non-clinically significant disturbances in heart rhythm were observed. | Ignored data dependency |
| Novodvorsky et al. ^19^ | Bradycardia was more frequent during nocturnal hypoglycaemia compared with matched euglycemia (incident rate ratio [IRR] 6.44 [95% CI 6.26, 6.63], p < 0.001). During daytime hypoglycaemia, bradycardia was less frequent (IRR 0.023 [95% CI 0.002, 0.26], p = 0.002) and atrial ectopics more frequent (IRR 2.29 [95% CI 1.19, 4.39], p = 0.013). | Aggregated |
| Middleton et al. ^20^ | Higher QT dynamicity, a poor prognostic factor in cardiac disease, was seen in participants who experienced hypoglycaemia compared with those who did not (0.193 vs. 0.159 for the nocturnal period; p = 0.01). | Aggregated |
| Desouza et al.^21^ | The difference between the frequency of ischaemia during hypoglycaemia and the frequency during both hyperglycaemia and normoglycemia was statistically significant (p *<* 0.01). | Ignored data dependency and aggregated |
| Kubiak et al. ^17^ | Glucose levels during night-time were negatively associated with QTc interval length if the data structure was accounted for (b [SE]= - 0.76 [0.17], (p *<* 0.01), that is 0.76ms decrease in mean QTc per 1 mmol/l increase in glucose. | Correct analysis |
| Cypryk et al. ^22^ | Higher glycaemia correlated with foetal heart rate (r = 0.32; p < 0.0001) and was associated with higher odds of the foetus developing small accelerations (odds ratio = 1.05; 95% confidence interval, 1.00–1.10; p = 0.04). | Ignored data dependency and correct analysis |
| Gill et al. ^23^ | Corrected QT interval (QTc) was longer during nocturnal hypoglycaemia compared with normoglycemic control periods (445±40 vs 415±23 ms; p = 0.037). | Ignored data dependency |
| Koivikko et al. ^24^ | The QTc interval (439 ± 5 vs. 373 ± 5 ms, respectively, p = 0.025) decreased significantly during hypoglycaemia. | Ignored data dependency and aggregated |
| Pistrosch et al. ^25^ | Participants with hypoglycaemic episodes had a significantly higher number of severe ventricular arrhythmias [ventricular tachycardia (VT) 32.8 ± 60 vs. 0.9 ± 4.2, p = 0.019]. | Aggregated |
| Koivikko et al. ^26^ | Hypoglycaemia resulted in significant decrease in the low frequency component of HR variability (2.134±1.965 vs 1.169 ± 1.029ms^2^ respectively, p = 0.006). | Aggregated |
| Borgognoni et al. ^16^ | There is a reduction of the total autonomic system activity in all diabetic patients compared with the control group expressed as a decrease of the SDNN (deviation of all  sinus rhythm RR intervals) values (DM: 126 ± 22.4 ms v control group: 176.1 ± 26.7 ms, p < 0.001). | Aggregated |
| Lee et al. ^27^ | There was a statistically significant prolongation of QTc during hypoglycaemia compared with that during euglycaemia (p = 0.035) and no significant difference in QTc during hyperglycaemia compared with euglycaemia (p = 0.470). | Ignored data dependency |
| Matsushita et al. ^28^ | In T2D, the fluctuation in fasting sympathetic nerve activity around wake-up was positively associated with not only morning but also daily GV. Delta Δ(the difference between the maximum and minimum value during 1 h before and after wake-up time, before breakfast.) of low frequency/high frequency (LF/HF) around wake-up time (Δ LF/HF wake-up) as positively associated with Δ glucose wake-up (b = 0.47, p = 0.011), standard deviation (SD) glucose wake-up (b = 0.48, p = 0.009), the mean amplitude of glucose excursions (MAGE24h) (b = 0.54, p = 0.002), and SD glucose24h (b = 0.41, p = 0.0025) after adjustment for age, sex, BMI, the duration of diabetes, and the prevalence of diabetic polyneuropathy. | Aggregated |
| Bernjak et al. ^29^ | During daytime, there was no significant difference between mean QTc interval (404 ±2 ms vs 407 ±20 ms, p = 0.263), significant decrease of T-peak to T-end interval duration corrected for heart rate (TpTendc) (74.8 ±16.1ms vs 79.0 ±14.8ms, p = 0.033) and significant T-wave symmetry increase: (1.62 ±0.33 vs 1.50 ±0.39, p = 0.02) in hyperglycaemia vs euglycaemia. During night-time there was no difference in mean QTc interval (401 ±26 ms vs 404 ± 27 ms, p = .13) during hyperglycaemia vs euglycaemia. | Correct analysis |
| Stahn et al. ^30^ | Patients with severe hypoglycemia (n = 12) had a higher number of severe ventricular arrhythmias (ventricular couplets 41.7±81.8 vs. 5.5 ± 16.7; ventricular tachycardia 1.0 ± 1.9 vs. 0.1 ± 0.3) compared to patients without severe hypoglycaemia. No direct correlation could be found among different variables of glucose profile, corrected QT interval, and ventricular arrhythmias. | Aggregated |
| Richardson et al. ^31^ | There was no overall correlation between reduced heart rate variability (a marker of autonomic dysfunction) and hypoglycemic events (*r* = 0.12, *p* = 0.62). | Aggregated |
| Pertseva et al. ^32^ | The blood glucose range, regardless of HbA1c, positively correlates with HRV – relative hypersympathicotonia: LF/HF 24-h, r= 0.43 (p<0.05), LF/HF day r = 0.37 (p<0.05) and r = 0.38 LF/HF night. | Aggregated |
| Cichosz et al. ^33^ | The HRV parameter low frequency (LF) was significantly (p = 0.029) reduced during hypoglycemia. Changes in LF during hypoglycemia were not different between patients with and without CAN (cardiovascular autonomic neuropathy), -0.64 versus -0.48 log(LF) (p = 0.74). | Aggregated |
| Kalopita et al. ^34^ | HRV was inversely associated with the standard deviation of the mean interstitial tissue glucose (MITG) and with the 𝑀-value during the entire recording (𝑟: −0.29, p = 0.052; 𝑟: −0.30, p = 0.047, resp.) and during the night (𝑟: −0.29, p = 0.047; 𝑟: −0.31, p = 0.03, resp.) | Aggregated |
| Abdelhamid et al. ^35^ | Hypoglycemia was associated with greater risk of bradycardia but  did not affect atrial or ventricular ectopics, heart rate variability, or cardiac repolarization. Bradycardia was more frequent during hypoglycemia (IRR, 24 [95% CI, 12–49]; p < 0.001). Hyperglycaemia did not affect the frequency of arrhythmias when compared with euglycemia. | Correct analysis |
| Charamba et al. ^36^ | There was a significant positive association between QTc and time spent in hyperglycaemia. QTc was significantly longer during hyperglycaemia compared to normal glucose and hypoglycaemia (9.97, p = 0.0001) and (6.45, p = 0.001), respectively. | Correct analysis |
| Yang et al. ^37^ | Compared with insulin monotherapy, metformin add-on significantly increased multiple heart rate variability parameters. Significant SDANN and PNN50 increases were observed in the metformin group com-pared with the nonmetformin group (difference, 25.62ms (0.15 to 51.09), p = 0:049, and 10.14ms (3.74 to 16.55), p = 0.004, respectively). Metformin add-on treatment significantly increased the log- transformed values of HF power compared with insulin treatment alone (0.01 ± 0.35 vs. −0.19 ± 0.34ms −1, difference, 0.38ms-1 (0.09 to 0.66), p = 0.01), with a significant decrease in the LF/HF ratio in the metformin group compared with that in the non metformin group (−0.27 ± 0.26 vs. 0.16 ± 0.36, difference, -0.47 (-0.75, -0.18), p = 0.006). | Aggregated |
| Abobarin‑Adeagbo et al. ^38^ | Group‑3 (symptomatic hypoglycemia) patients had more documented hypoglycemic episodes (0.8 ± 0.5 per 24 h) than Group‑2 (hypertensive crisis present) patients (0.2 ± 0.3 per 24 h), however, they were not different to the ones in Group‑1 patients (0.4 ± 0.4 per 24 h). Plasma norepinephrine and mean arterial blood pressure were higher Group‑1 (insulin‑treated patients) and Group‑3 patients than in control patients of Group 2. At discharge, the daily cumulative insulin dose was reduced in Group‑1 (− 18.4 ± 24.9 units) and in Group‑3 patients (− 18.6 ± 22.7 units), but remained unchanged in Group‑2 control patients (− 2.9 ± 15.6 units). | Aggregated |
